# Microneedles from Fishscale-Nanocellulose Blends Using Low Temperature Mechanical Press Method

**DOI:** 10.3390/pharmaceutics7040363

**Published:** 2015-09-24

**Authors:** Ololade Olatunji, Richard T. Olsson

**Affiliations:** 1 Chemical Engineering Department, University of Lagos, Akoka, Lagos, Nigeria; 2 Fibre and Polymer Technology, School of Chemical Science and Engineering, KTH–Royal Institute of Technology, SE-100-44 Stockholm, Sweden; E-Mail: rols@KTH.se

**Keywords:** microneedles, nanoparticles, cellulose, fish scale, biopolymer, drug delivery, biomaterials, micromolding

## Abstract

Fish scale biopolymer blended with nanocellulose crystals is used for production of microneedles applying mechanical press microfabrication and the effect of nanocellulose on microfabrication, water absorption, moisture stability and mechanical properties of the microneedles is reported. The results show that microneedles produced from the nanocellulose loaded fish scale biopolymer requires higher temperature for micromolding (80 ± 5 °C) than microneedles from only fish scale biopolymer, which were moldable at 50 ± 5 °C. The mechanical properties of the fish scale biopolymer-nanocellulose (FSBP-NC) films showed that the addition of nanocellulose (NC) resulted in lower elongation and higher tensile stress compared to fish scale biopolymer (FSBP) films. The nanocellulose also prevented dissolution of the needles and absorbed up to 300% and 234% its own weight in water (8% and 12% *w*/*w* NC/FSBP), whereas FSBP films dissolved completely within 1 min, Indicating that the FSBP-NC films can be used to produce microneedles with prolonged dissolution rate. FTIR spectrometry of the FSBP films was compared with the FSBP-NC films and the NC gels. The FTIR showed typical peaks for fish scale polymer and nanocellulose with evidence of interactions. SEM micrographs showed relatively good dispersion of NC in FSBP at both NC contents corresponding to 8% and 12% *w*/*w* NC/FSBP respectively.

## 1. Introduction

Microneedle patches used for biomedical applications such as transdermal drug delivery and vaccine delivery are produced from materials that not only need to be biodegradable and biocompatible but must also be processed using reproducible microfabrication techniques and be strong enough to pierce into the skin. The feasibility of biopolymers extracted from fish scales for production of microneedles have been presented and showed that structures with sharp tips were successfully formed and effectively pierced and dissolved into the skin under 1 min [1]. The method that is commonly used for the production of dissolvable polymer microneedles is the centrifugation method [1,2,3,4]. The centrifuge method requires up to about 15 min centrifugation time in addition to several hours of drying at room temperature, to ensure complete filling and consolidation of the polymer inside the template cavities. An alternative method would be to use a mechanical press for the micromolding. The mechanical press method was previously used in the production of microneedles from polycarbonate and involved heating of polycarbonate close to 200 °C to obtain out of plane microneedles with sharp tips in relatively short times [5]. It has not yet been demonstrated for microfabrication of biopolymer microneedles and would present the benefit of requiring shorter times for microfabrication.

A major challenge within the context of preparing biodegradable polymeric microneedles is the known property of biopolymers such as gelatin, collagen, and starch is the tendency to absorb water, which also affects their stability post processing [6,7,8]. Despite the challenge posed on the stability of biodegradable polymer due to water absorption, little research has been directed towards enhancing the stability of biodegradable polymer based microneedles by using natural filler reinforcements such as cellulose. Cellulose nanoparticles have been applied in high toughness membranes for their mechanical strength [9] they also have biocompatible properties and have been used in biomedical applications such as drug delivery and scaffolds [10,11]. The tendency of cellulose to absorb water is undesired in hydrophobic engineering polymers as this leads to weakening of the fibre and composite [10,12,13]. Efforts to address this includes: limiting the cellulose content within the composite to the minimal amount required to improve mechanical strength [13], improving the bonding between the fibre and matrix using a compatibilizer [6] or alternatively by modifying the cellulose fibres to be more hydrophobic [12]. However, at the same time hydrophilic polymers are known to perform better due to their improved miscibility with cellulose. For example unmodified ramie cellulose reduced the water sensitivity of starch composite compared to the neat starch polymer [14].

In this study, we apply the mechanical press microfabrication in the production of reproducible biodegradable microneedles form fish scale biopolymer reinforced with nanocellulose (FSBP-NC). We look at applying this method at a much lower temperature, which requires less waiting time in addition to the possibility of preparing arrays of microneedles from FSBP based materials. To this effect this study also looks at the effect of nanocellulose (NC) on the fabrication, mechanical strength and moisture stability of fish scale microneedles. It is demonstrated that the nanocellulose allows stiffer and stronger needles to be produced, with the benefit of improved moisture stability.

## 2. Experimental Section

### 2.1. Materials

Fish scales were obtained from a fish trader in Ejigbo market in Lagos, Nigeria. The trader mainly sells tilapia and only tilapia scales were collected for this study. The scales were then taken to the laboratory where they were washed repeatedly to remove all debris, skin and dirt. They were then kept in the freezer at −20 °C until when needed. The wood pulp was delivered by Nordic Paper, Stockholm, Sweden. Silicone elastomer (Sylgard 184i9) was obtained from Sigma–Aldrich, London, UK. Capillary tubes (Pyrex, Stuart, 0.2 mm i.d.) were purchased from Finlab, Lagos, Nigeria. Stainless steel microneedles Admin Patch were purchased from Admin Med, Sunnyvale, CA, USA. The patches were made up of 1500 µm microneedles array containing 31 microneedles arranges on a 1 cm^2^ area array made of stainless steel of medical grade. Polycarbonate microneedles were obtained from MITI systems, Seoul, Korea. Microneedles were solid tapered shape with a height of 500 µm on 30 mm × 15 mm sheets containing 176 microneedles.

### 2.2. Production of Fish Scale Biopolymer Films

Fish scale biopolymers was extracted according to method reported in previous studies Olatunji *et al.* [1]. One hundred grams of thawed fish scale was weighed and treated with 0.6% *w*/*v* sodium hydroxide to remove non collagen residues from the fish scales. Sodium hydroxide pre-treatment was carried out at normal room temperature for a duration of 1 h while swirling every 15 min in a conical flask.

Following the pre-treatment stage, the scales were washed several times using tap water until the sodium hydroxide was completely removed. The scales were then transferred into the stainless steel pressure vessel of 4.5 L capacity (PC-70001, Binatone, Lagos, Nigeria) containing 200 mL of water. The vessel is closed and heated at 80 °C (±3 °C) for 8 h at a pressure of 80 kPa. At the applied conditions, the collagen is hydrolysed and dissolves in the water [15]. On completion of the extraction stage, the liquid is separated from the solid using a 2 mm aperture sieve and further separation using a centrifuge (Corning LSE Compact centrifuge, Sigma–Aldrich) at 3500 rpm for 20 min. Customized silicone plates were prepared using silicone elastomer and activator (Polycraft T4 Silastic, MBFG, Newtownabbey, UK) in the ratio 10:1 by weight. The supernatant was separated and transferred unto the customized silicone plate to oven dry at 150 °C for 1 h at atmospheric pressure allowing the water to evaporate. The films resulting were then left to air dry on the silicone plate at room temperature for 24 h to form dry films. The films were removed from the silicone plates manually and stored at room temperature (30 ± 10 °C) until next use.

### 2.3. Production of NC Gel

Wood pulp with hemicellulose and lignin contents of 13.8% and 0.7% was used as raw material for the extraction of TEMPO-oxidized nanocellulose. Forty grams of pulp was suspended in 4 L of water, containing 0.64 g of oxidation mediator 2,2,6,6-tetramethylpiperidinyl-1-oxyl (TEMPO) and 4 g of sodium bromide. The oxidation was carried out by adding 2 mol/L NaClO (10 mmol per gram of cellulose) to the suspension. The pH of the suspension was maintained at 10 by adding 0.5 mol/L NaOH with a pH stat for 5 h. The reaction was then quenched by addition of 0.2 L ethanol. The resulting suspensions were then washed with water and isothermally centrifuged at 14,000 rpm on a Sorvall RC 5C Plus Centrifuge (Bekman Coulter, Brea, CA, USA). The resulting nanocellulose was characterized by TEM, SEM and AFM *etc.* in [16].

### 2.4. Production of FSBP-NC Films

To prepare FSBP-NC films, 5 g FSBP film was dissolved in 10 mL of distilled water at 45 °C. Ten milliliter of water was added to 20 and 30 g of NC and stirred for 1 h at room temperature (27 ± 3 °C) to obtain a uniform gel. The dissolved FSBP was then added to the gel drop by drop. The mixture was stirred for 1 h at 200 rpm at room temperature on a magnetic stirrer (IKA^®^ CMag HS4, from Sigma–Aldrich), the temperature was then increased to 80 °C and the mixture allowed drying while stirring until a thick gel is formed. The thick gel was then transferred unto a silicone plate and allowed to dry at room temperature for 24 h.

### 2.5. Fabrication of Microneedles

In this study, both single and arrays of microneedles were produced. Single microneedles were produced from tips of pulled capillary tubes using method reported by Olatunji *et al.* [1]. Arrays of microneedles were produced using established micromolding techniques [2]. However, in this study, we make a further modification to this method by using a heat press machine in place of centrifuge, which is commonly used for biodegradable polymer microneedles.

To produce array microneedles, silicone elastomer was poured over solid microneedle molds and left to cure for 24 h. The solid mold was then separated from the silicone mold. For single microneedles glass capillary tubes were pulled on a Bunsen burner to form sharp tips. The tips were characterized using digital imaging at 15× magnification to view tips formed. The formed tips were then separated from the rest of the capillary tube by breaking off with tweezers and attaching to flat bases of the fish scale polymer film which was slightly wetted with water to aid adhesion. Silicone elastomer mixed in the ratio 10:1 (weight of silicone to activator) was poured over the glass microneedle master mold and allowed to set for about 24 h after which the silicone mold is separated from the glass master mold.

To produce either single or array of microneedles, sample of the films were cut with scissors and placed on the silicone microneedle mold. A metal sheet is used to cover the films and the sample placed between two metal blocks of the heat press machine.

A temperature of 50, 60, 70, 80, 90 °C and constant pressure of 10 N/cm^2^ is applied and held at this condition for 10 min. The temperature is then turned off and allowed to cool for 5 min after which the mold with polymer is removed and allowed to cool for a further 5 min on cooling blocks. The formed microneedle is then separated from the silicone mold manually. The arrays and tips formed are observed using digital imaging.

### 2.6. Mechanical Properties of FSBP-NC Films

Mechanical properties (tensile strength, young modulus, elongation at break, and yield strength) were obtained using method as described in [1] the Thwing Albert tensile tester (Thwing Albert, West Berlin, NJ, USA). The films were cut into equal rectangular strips of 2 cm width. The film thickness is obtained by taking average of the thickness of the films at six different points along the length using a micrometer. The films were placed between the two grips of the force analyzer and set at an initial distance of 5 cm between the two grips thus representing the length over which the tensile stress is exerted. At a set speed of 3 cm/min the film was pulled in opposite direction by the two grips and a graph of force against displacement was obtained using the data logger on the force analyzer. The room temperature ranged between 25 and 30 °C over the period of testing.

### 2.7. Water Absorption Capacity

To study the behavior of the FSBP-NC in water, the method used by [17] was adopted. One gram of FSBP-NC and FSBP films of the same thickness and dimension were placed into two separate test tubes containing 10 mL of distilled water. The films were removed and weighed after different time intervals of 10 min. The time for the films to completely dissolve was also recorded.

### 2.8. Fracture Force of Microneedles

To measure force required to fracture microneedles, single microneedles were employed rather than using an array containing several microneedles as it would be more difficult to view the fracture point. The single microneedle is placed on a flat adhesive substrate such that the base is firmly fixed to the force tester machine (Thwing albert). Compressive force is then applied at a speed of 5 mm/s. A sharp drop in the force recorded indicates the fracture point. The tips were then observed with digital imaging to confirm the fracture. This was done for FSBP-NC and FSBP microneedles.

### 2.9. FTIR Spectroscopy

To obtain FTIR peaks for each films, a diamond crystal ATR spectrometer accessory attached to the Agilent cary 630 FTIR was used at a wavelength ranging between 4000–500 cm^−1^. The sample of NC gel, FSBP-NC and FSBP films were placed on the sample window and the press closed to allow contact. The sample was then scanned to obtain the absorbance/transmittance plot.

### 2.10. Moisture Content Analysis

The moisture content of the films was measured using a moisture content analyzer (MS-70, AND, Birmingham, UK). One gram of sample from the films was placed on a steel base which was placed on the sample port of the moisture analyzer, the sample was heated at 180 °C (this temperature was based on the preliminary analysis on the moisture analyzer which indicates this as the optimal temperature for the material) until all moisture content was removed from the sample. The value of the moisture content was then recorded and the experiment stopped. The moisture analysis was performed at the same temperature for all film samples used in this study and each sample was determined as average of three repeated measurements.

### 2.11. Microscopy

Scanning electron microscopy (SEM) was performed on a Hitachi S-4800, after 40 s Pt/Pd sputtering. The cross-sectional surfaces of cryo-fractured casted films were vertically mounted on aluminum stubs by immersing half of the 3 mm wide film samples into liquid carbon paste, which further was allowed to dry for 24 h before observation. The microscope was operated at a working distance (WD) of 4 mm.

### 2.12. Skin Insertion Studies

Microneedles were inserted into porcine skin cut out from the ear of slaughtered pigs obtained from “Odo” market in Lagos. The pig ear was collected from the butchers on the same day as slaughtered, and taken to the laboratory where it was washed and hair removed using a shaving stick. Microneedles were inserted by hand and immediately removed after which methylene blue solution was applied to the pierced area, left for 1 h and excess solution was wiped off with clean tissue. The area was then imaged with digital camera to observe if skin had been pierced.

## 3. Results and Discussion

Visual observation of the fish scale biopolymer nanocellulose (FSBP-NC) films showed uniform films with apparent good blend of the NC gel and FSBP which indicated the absence of phase separation during the mixing. At room temperature, the films of the FSBP-NC appeared more rigid while the FSBP films were more flexible. In order to obtain a more complete understanding for the location of the cellulose phase, the films were frozen in liquid nitrogen and cryo-fractured before scanning electron microscopy (SEM) examination.

### 3.1. Scanning Electron Microscopy (SEM) Imaging

**Figure 1:**
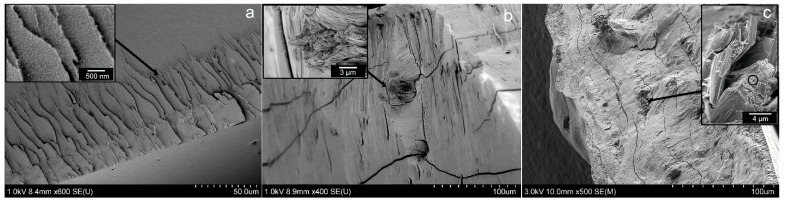
SEM images of (**a**) FSBP (**b**) NC 20 (**c**) NC 30 films, with insets highlighting the characteristics of the surface morphologies.

Figure 1a shows the pristine films of FSBP, which fracture in a homogenous manner, leaving a completely smooth interface on micrometer level magnification. Only a grainy surface pattern could be observed at higher magnification due to an insignificant plastic yielding on the “flake-like” areas visible in the inset of Figure 1a. Overall the film forming properties were very good.

**Figure 2:**
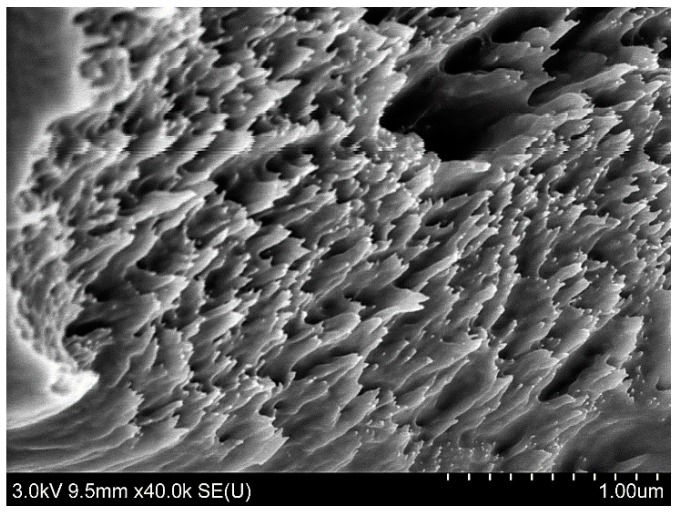
SEM image of the NC 30 film containing 12 wt % (highlighted area in circle Figure 1c) nanocellulose, showing the nanofibres of cellulose embedded inside the gelatine matrix.

The SEM images of the films containing nanocellulose (Figure 1b,c) showed a fairly good dispersion of the NC in the FSBP films considering the relatively large contents of cellulose used. However, the insets in Figure 1b,c also show regions that were segregated from the gelatine host matrix, especially for the 30 g sample (Figure 1c inset). These regions could be confirmed to contain the thin cellulose fibrils, see Figure 1c inset. The circle marked area in the inset of Figure 1c is displayed in Figure 2, where the individual cellulose nanofibers can be seen. It was therefore concluded that an upper limit for inclusion of the cellulose with carboxylic groups on its surface, was *ca.* 20 wt % (Figure 1b). Above this filler level, the cellulose tended to self-associate into more concentrated regions, which are visible as protruding sections from the cross-sectional crack in Figure 1c.

### 3.2. Mechanical Properties of Films

**Figure 3:**
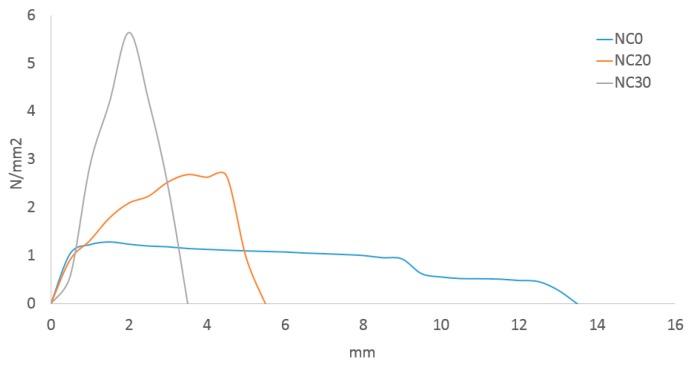
Stress strain graph for fish scale biopolymer films loaded with 0, 20 and 30 g nanocellulose gel (0%, 8% and 12% *w*/*w* cellulose nanoparticles).

**Figure 4:**
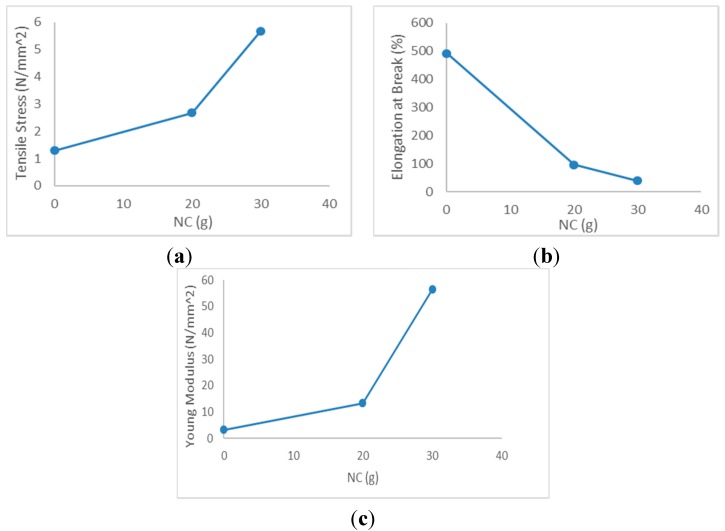
(**a**) Tensile stress, (**b**) elongation at break, and (**c**) young modulus for different FSBP-NC.

Figure 3 shows the average stress strain graph of FSBP-NC and Figure 4 shows the tensile stress, elongation at break and young modulus. Results obtained showed that the tensile stress and young modulus were higher for FSBP-NC than FSBP. Increasing the NC content from 20 to 30 g increased the tensile stress and young modulus from 2.67 to 5.65 N/mm^2^ and from 13.35 to 56.5 N/mm^2^, showing that the inclusion of NC had a marked effect on the toughness of the FSBP. Whereas the strength increased *ca.* 10% and 33%, the toughness decreased *ca.* 20% for the FSBP-NC composites as the elongation at break was much higher for FSBP films than for FSBP-NC films, decreasing from 492% to 40% for films containing 30 g of NC gel.

The reduced elongation at break with respect to NC concentration was likely due to the slightly phase separated FSBP-NC blend, which would be in accordance with the microscopy results. Water has a plasticizing effect on the FSBP, the reduction in elongation at break for films containing NC was possibly also attributed to a reduced moisture content in the FSBP phase, which occurred during drying due to the moisture stabilizing/attracting ability of the nanocellulose.

### 3.3. FTIR Spectroscopy

**Figure 5:**
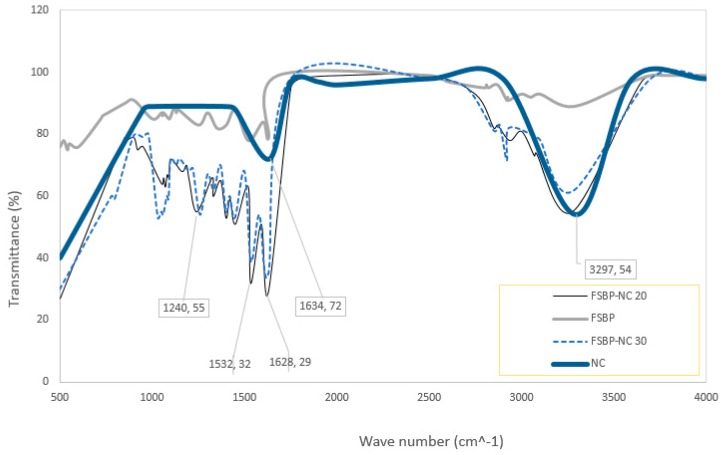
FTIR of NC and FSBP with 20 and 30 g NC gel.

The FTIR spec for the nanocellulose gel and fish scale biopolymer with and without NC is shown in Figure 5. Typical peaks for NC and FSBP are seen, amide A bond at wavelength of about 3200 cm^−1^, amide I, II and III bond at about 1600, 1500 and 1200 cm^−1^ respectively. The peaks obtained here are similar to those obtained for gelatine from fish skins and bones and the nanocellulose. Thus, confirming that the biopolymer extracted from the scales of fish is gelatine. Furthermore, the increase in intensity at 3200 cm^−1^ indicates presence of moisture as the cellulose absorbs the water from the gelatine, which is thought to cause the decreased elongation at break seen for films with NC. The decreased intensities of the amide I, II and III bonds is likely due to the interactions of the cellulose and amide or the protein dilution effects caused by the addition of NC. These observations are in accordance with those from the FTIR analysis of similar blends of gelatine and cellulose [8,18].

### 3.4. Water Absorption Capacity

**Figure 6:**
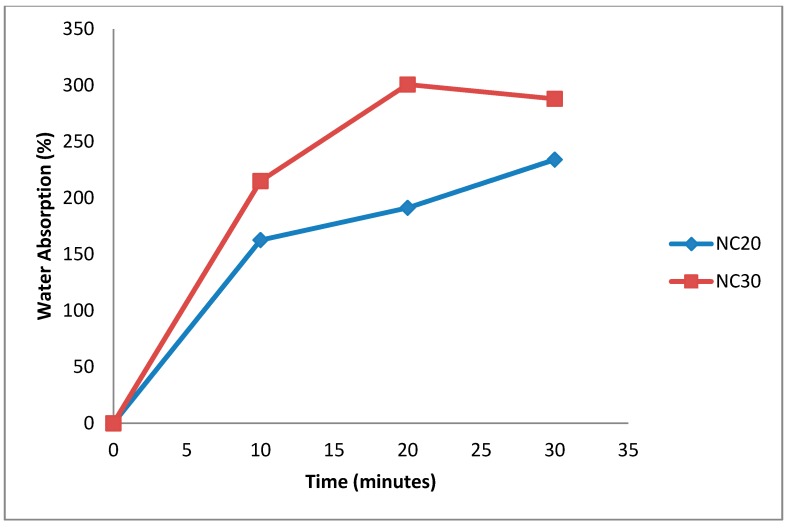
Moisture absorption of Fish scale biopolymer with 20 g NC/5 g FSBP and 30 g NC/5 g FSBP nanocellulose gel.

**Figure 7:**
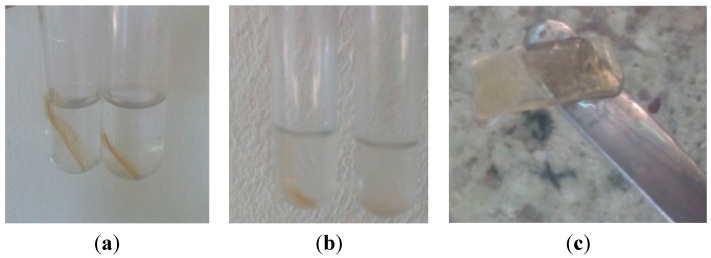
Films with and without nanocellulose (**a**) at time 0 and (**b**) after 20 min in distilled water shows FSBP-NC film still undissolved while FSBP films had completely dispersed in water (**c**) films with nanocellulose after 30 min in water still maintain shape (albeit swollen haven absorbed water).

The FSBP-NC films had more water absorption capacity as they increased up to 0.4 g in weight for NC 30 and 0.334 g for NC 20 (Figure 6). For the FSBP-NC films it was necessary to leave them overnight to allow films to completely degrade in water. As shown in Figure 7 even after 30 min in water the FSBP-NC films still had sufficient strength to be held on a spatula without disintegrating. The weight decrease occurred after about 35 min as the films began to disintegrate (slowly). This was taken to be the limit for water absorption. A further 12 h was required for the FSBP-NC films to completely degraded in water whereas the FSBP films completely dissolved in water in less than 5 min. From this result it is evident that albeit the SEM points to some degree of aggregation of the cellulose nanoparticles as shown by appearance of tree-like protruding structures, the cellulose nanoparticles were sufficiently dispersed within the fish scale biopolymer to have notable effect on water absorption and its ability to retain some mechanical properties as a dispersed network of cellulose nanofibres.

This NC related water absorption capacity is a desirable property that could be useful in developing microneedles with controlled water absorption properties. This will be further explored in our future studies.

### 3.5. Moisture Content

**Figure 8:**
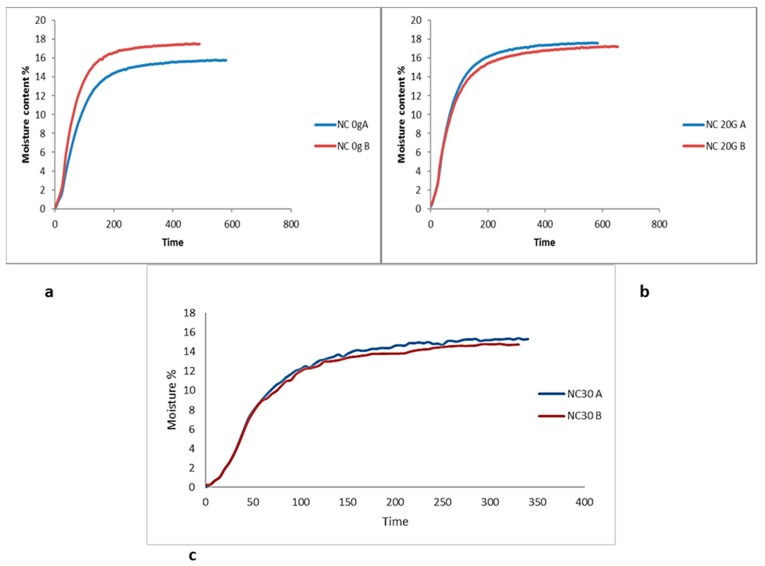
Moisture profile of fish scale biopolymer films containing (**a**) 0 g, (**b**) 20 g, and (**c**) 30 g nanocellulose.

The moisture content following drying for 24 h did not seem to vary significantly between the films (Figure 8). However some variations in moisture content was observed after the films were left in open air for 1 week. Films containing NC show less variation in moisture content over time thus indicating improved moisture stability, which is suggested to stem from the TEMPO oxidized cellulose surface strong ability to retain associated water. Whereas for all repeated measurements on films of FSBP showed a significantly greater difference in moisture content was observed after 1 week.

### 3.6. Microneedle Production

All films used for microneedle production were used shortly after drying such that they maintain about 16%–18% moisture content. Previous studies showed that glass transition temperature of biopolymers such as fish gelatin, wheat gluten, heparin, proteins and polyamides is dependent on moisture content and actual glass transition temperature cannot easily be measured using typical methods [19]. The measured glass transition temperature for fish gelatin ranged between 40 and 190 °C [7]. Therefore we expect that microneedles will be more easily formed using the mechanical heat press method if moisture content is increased. This may however pose additional challenges such as breakage or bending of microneedles on removal from mold due to the increased moisture content resulting in weaker structure. In this study, the microneedles were already dry upon removal.

**Figure 9:**
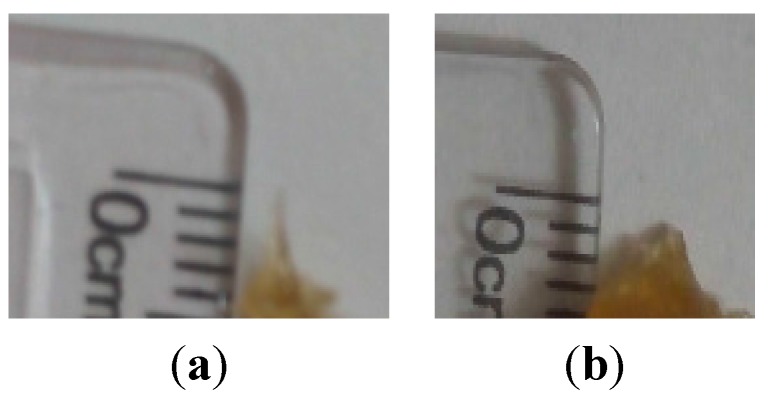
Microneedles formed using micromolding at 70 °C for (**a**) Fish scale biopolymer (**b**) Fish scale biopolymer with 20 g nanocellulose.

**Figure 10:**
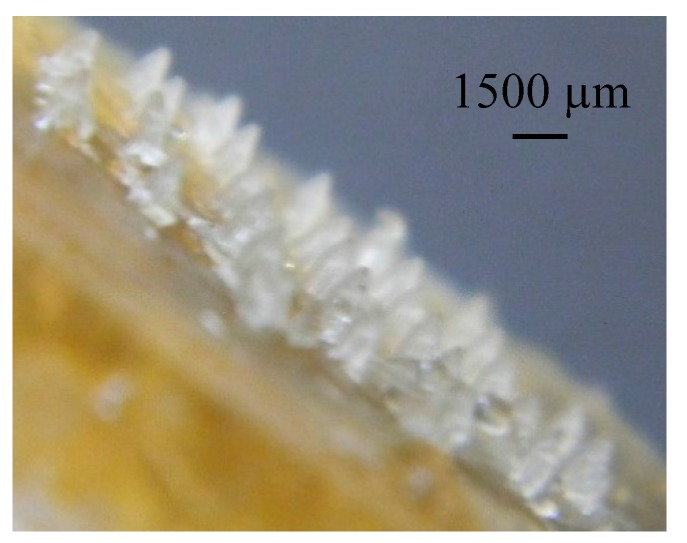
1500 µm-long microneedle array formed from FSBP at 50 °C.

**Figure 11:**
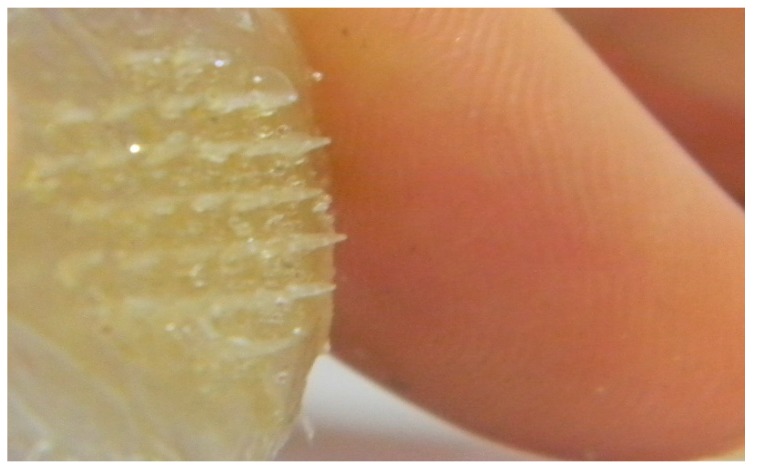
Microneedle array formed from FSBP-NC 20 g at 80 °C placed next to a human finger.

**Figure 12:**
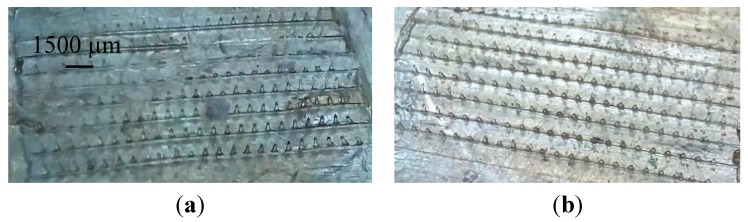
Digital images of (**a**) NC 20 g and (**b**) FSBP microneedles formed at 70 °C.

Figure 9, Figure 10 and Figure 11 shows single and arrays of microneedles formed from FSBP-NC and FSBP films that were made using the low temperature press method. The single and arrays of microneedles were formed at temperature between 50 and 90 °C. At 70 °C FSBP successfully formed both array and single microneedles while FSBP-NC only partially formed needles, see (Figure 12). As seen in Figure 9, the tippets of the FSBP microneedle was much sharper than that of FSBP-NC. It was necessary to increase the temperature to 90 °C to obtain complete array for FSBP-NC films for the NC20. This was likely due to the fact that the FSBP-NC films showed lower elasticity at 70 °C than the FSBP films as shown in Figure 4b.

At the pressure used, microneedles did not form for NC 30 which was likely due to further reduction in plasticity of the polymer such that the plasticity required for micromolding is not reached for NC 30. Perhaps increasing the pressure would result in micromolding for NC 30; however, due to the limitation of our equipment, pressure was not varied in this study.

To ensure that the method of fabrication of dissolvable microneedles is applicable to other geometries, arrays of microneedles were also produced. These microneedles 300 mm in length with 184 parallel microneedles in an array are shown in Figure 12. Figure 12 shows FSBP-NC and FSBP microneedles formed at 70 °C. The FSBP-NC (NC 20) microneedle arrays only partially formed while the FSBP array was fully formed.

### 3.7. Fracture Force on Microneedles

**Figure 13:**
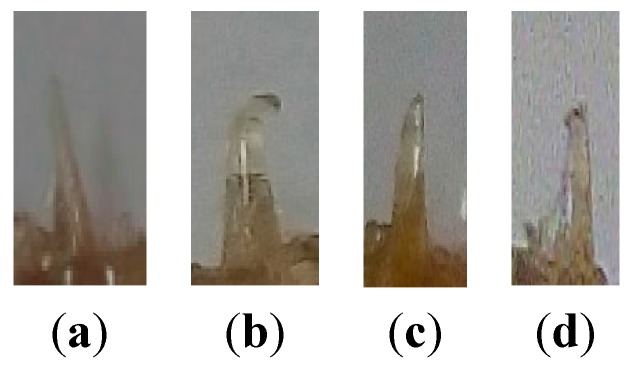
NC 0 (**a**) before fracture and (**b**) after fracture after 0.04 N, (**c**) NC 20 before fracture and (**d**) fractured after 0.5 N.

For ease of visualization and measurement, single microneedles were employed in the study of fracture force of microneedles. The microneedles produced from FSBP-NC required a force of up to 0.5 N to fracture while those from FSBP fractured after a load of 0.04 N was applied. Microneedles from fish scale microneedles with tip radius of about 40 mm were able to withstand force of up to 0.02 N but microneedles broke at 0.04 N (Figure 13).

### 3.8. Skin Insertion Studies

**Figure 14:**
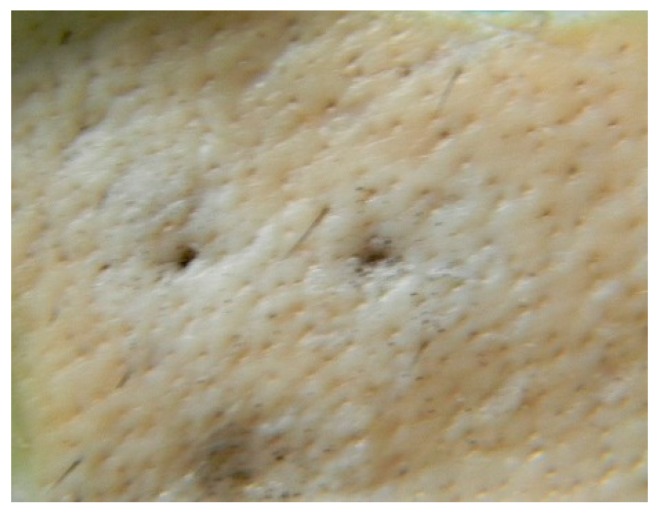
Porcine skin pierced with FSBP (left) and NC (right) single microneedle.

When inserted into the skin, both single and array microneedles made from FSBP-NC and FSBP pierced porcine skin successfully. Figure 14 shows pierced porcine skin after microneedle insertion and staining with methylene blue and cleaning. In most cases a few trials with different microneedles were necessary to successfully obtain observable piercing on skin. The insertion test carried out in this study indicates that the microneedles have sufficient strength to pierce the upper layer of the skin. More advanced techniques can be applied to obtain further information on the characterization of the holes created by microneedles in the skin, however this is not within the scope of this work.

## 4. Conclusions

The application of FSBP-NC films for biopolymer microneedle production resulted in microneedles with sufficient strength to penetrate the skin. The films had sufficient flexibility and thermal property for micromolding, particularly films containing 20 g NC. This studies shows that consistent arrays of microneedles can be produced from biodegradable polymers of FSBP and NC blends using the mechanical press method at relatively low temperatures. Despite some degree of aggregation seen in the SEM imaging, the dispersion of the NC in the FSBP is sufficient to affect the mechanical and moisture properties as shown in the results discussed. The water absorption capacity and moisture stability is significantly increased for films containing 20 and 30 g NC FSBP. The combined effect of these properties results in FSBP-NC microneedles which are strong enough to pierce into the skin and degrade at varying rates.
